# Complete Genome Sequence Analysis of *Ralstonia solanacearum* Strain PeaFJ1 Provides Insights Into Its Strong Virulence in Peanut Plants

**DOI:** 10.3389/fmicb.2022.830900

**Published:** 2022-02-23

**Authors:** Xiaodan Tan, Xiaoqiu Dai, Ting Chen, Yushuang Wu, Dong Yang, Yixiong Zheng, Huilan Chen, Xiaorong Wan, Yong Yang

**Affiliations:** ^1^Guangzhou Key Laboratory for Research and Development of Crop Germplasm Resources, Zhongkai University of Agriculture and Engineering, Guangzhou, China; ^2^Key Laboratory of Horticultural Plant Biology (HZAU), Ministry of Education, Key Laboratory of Potato Biology and Biotechnology (HZAU), Ministry of Agriculture and Rural Affairs, Huazhong Agricultural University, Wuhan, China

**Keywords:** *Ralstonia solanacearum*, peanut, genome sequencing, comparative genomic analysis, pathogenicity

## Abstract

The bacterial wilt of peanut (*Arachis hypogaea* L.) caused by *Ralstonia solanacearum* is a devastating soil-borne disease that seriously restricted the world peanut production. However, the molecular mechanism of *R. solanacearum–*peanut interaction remains largely unknown. We found that *R. solanacearum* HA4-1 and PeaFJ1 isolated from peanut plants showed different pathogenicity by inoculating more than 110 cultivated peanuts. Phylogenetic tree analysis demonstrated that HA4-1 and PeaFJ1 both belonged to phylotype I and sequevar 14M, which indicates a high degree of genomic homology between them. Genomic sequencing and comparative genomic analysis of PeaFJ1 revealed 153 strain-specific genes compared with HA4-1. The PeaFJ1 strain-specific genes consisted of diverse virulence-related genes including LysR-type transcriptional regulators, two-component system-related genes, and genes contributing to motility and adhesion. In addition, the repertoire of the type III effectors of PeaFJ1 was bioinformatically compared with that of HA4-1 to find the candidate effectors responsible for their different virulences. There are 79 effectors in the PeaFJ1 genome, only 4 of which are different effectors compared with HA4-1, including RipS4, RipBB, RipBS, and RS_T3E_Hyp6. Based on the virulence profiles of the two strains against peanuts, we speculated that RipS4 and RipBB are candidate virulence effectors in PeaFJ1 while RipBS and RS_T3E_Hyp6 are avirulence effectors in HA4-1. In general, our research greatly reduced the scope of virulence-related genes and made it easier to find out the candidates that caused the difference in pathogenicity between the two strains. These results will help to reveal the molecular mechanism of peanut–*R. solanacearum* interaction and develop targeted control strategies in the future.

## Introduction

Cultivated peanut (*Arachis hypoaea* L.), also known as groundnut, is a major oilseed legume crop grown in more than 100 countries around the world. It is often consumed in different forms of high-quality edible oil, edible nuts, peanut jelly, and sweets (Pandey et al., 2012). Approximately 44 million tons of peanuts are produced and consumed annually all over the world (FAOSTAT, 2016^1^). China is the largest peanut producer in total annual production, accounting for about 40.26% in the world. However, bacterial wilt, a destructive disease caused by *Ralstonia solanacearum*, always seriously blocks the peanut production all over the world. It outbreaks in the 13 main peanut-producing provinces of China and causes up to 50–100% yield losses (Jiang et al., 2017). Although bacterial wilt has been well studied in other plants, there are few studies in peanut, and current research mainly focused on transcriptome change after *R. solanacearum* inoculation and resistance marker screening (Chen et al., 2014; Luo et al., 2019). Up to now, some quantitative trait locus (QTLs) and resistance genes (e.g., *AhRLK1*, *AhRRS5*, and *AhGLKb*) against bacterial wilt have been identified in peanut (Zhang et al., 2017, 2019; Ali et al., 2020), but the molecular mechanism of peanut–*R. solanacearum* interaction is still poorly understood. The main reasons for this are that the peanut plant itself is not easy to conduct molecular experiments with, and it is the lack of specialized and in-depth research on the pathogenic bacteria causing peanut bacterial wilt.

*R. solanacearum* strains, by sensing root exudates, move to host roots using chemotaxis and flagellar movement (Yao and Allen, 2006; Hida et al., 2015). They enter the root through wounds, root tips, and secondary root emerging points; move to the vascular bundle; and reach the vascular tissue. Once inside the vascular system, the bacteria will quickly spread throughout the host’s body. Some bacteria are planktonic in the vascular fluid stream, while others use twitching motility to move along the vascular wall (Liu et al., 2001). These isolated cells eventually grow into biofilm-dependent aggregates that fill the entire vascular system and block water flow (Tran et al., 2016; Caldwell et al., 2017). In summary, the bacteria can break through the root system of the plant, enter the vascular system, multiply in the host body, and cause irreversible wilting of the plant.

The pathogenicity of *R. solanacearum* is the result of the cooperation and coordination of various pathogenic factors, mainly including extracellular polysaccharide (EPS), type II secretion system (T2SS), and type III secretion system (T3SS). After *R. solanacearum* enters the vascular system of plants, it will secrete a large number of EPS to block the vascular bundle and eventually cause the wilting of plants. T2SS can secrete cell wall degradation enzymes, cellulose, and pectin enzymes and produce motion and attachment elements and chemotaxis, which play key roles in the pathogenicity of *R. solanacearum* (Genin and Denny, 2012). T3SS can secrete type III effectors (T3Es), which play an important role in the pathogenesis of susceptible plants and the hypersensitive response of resistant plants. At present, the total number of T3Es identified in *R. solanacearum* complex species is about 120 (each strain varies greatly), which is significantly more than other plant-pathogenic bacteria (such as Pseudomonas and Xanthomonas) (Peeters et al., 2013). There is a large degree of polymorphism and functional redundancy in the T3Es of *R. solanacearum*, which is an important reason for its wide host range and host specificity (Macho et al., 2010). T3Es can help pathogens infecting the host as virulence proteins to inhibit the immune system. On the other hand, some T3Es can be recognized by the resistant proteins of the host as avirulence proteins to activate downstream immune signaling pathways and trigger defense response (Coll and Valls, 2013).

Since the first *R. solanacearum* GMI1000 was sequenced (Salanoubat et al., 2002), more and more *R. solanacearum* genome information has been released. However, only two *R. solanacearum* strains from peanut have been sequenced (Tan et al., 2019; Chen et al., 2021). With the development of sequencing technology and discovery of new *R. solanacearum* isolates, comparative genome analysis played an important role in discovering the virulence and avirulence factors of many pathogens. Previously, several genomics comparison studies were performed in order to identify differences in the gene content of *R. solanacearum* corresponding to low or high virulence in different hosts. A deletion of 33.7 kb was found in the megaplasmid of strain UY043 isolated from soil by comparative genomics hybridization analysis (Cruz et al., 2014). This region contains a cluster of six genes involved in type IV pili synthesis, which contributes to early bacterial wilt pathogenesis and the colonization fitness of potato roots. Another case of *R. solanacearum* was reported for two sequenced phylotype IIB-1 strains IPO1609 and UW551. The two strains are closely related but differ significantly in the virulence in their host plants. The research revealed that IPO1609 carries a 77 kb genomic deletion, which is responsible for almost complete loss of pathogenicity of the strain (Gonzalez et al., 2011). Recently, the *R. solanacearum* avirulence effectors RipJ and RipAZ1 were identified by the comparative genomic analysis of two strains with different virulence against *Solanum pimpinellifolium* and *S. Americanum*, respectively (Moon et al., 2021; Pandey and Moon, 2021). In the current study, we performed a comprehensive comparative analysis of the genome sequences of *R. solanacearum* strain PeaFJ1 and other strains, especially HA4-1. PeaFJ1 and HA4-1 were isolated from peanut, and PeaFJ1 showed more aggressiveness in many cultivated peanut varieties than HA4-1. The genes that may be responsible for the hypervirulence of *R. solanacearum* strain PeaFJ1 were revealed and analyzed in this study.

## Materials and Methods

### Strain Information and Cultivation

*R. solanacearum* strain PeaFJ1 was isolated from wilt peanut plant in Fuzhou City, Fujian Province of China and was identified as phylotype I, sequevar 14M, biovar 3 (Wang et al., 2017). *R. solanacearum* stored in an ultra-cold storage freezer was revived by incubating on a casamino acid-peptone-glucose (CPG) solid medium containing triphenyltetrazolium chloride (TTC) (10 g/L peptone, 2.5 g/L glucose, 1 g/L casamino acids, 50 mg/L TTC, 15 g/L agar) at 28°C for 48–72 h. The pearly cream-white but displaying pink in the center, irregular shaped and fluidal colonies are typical *R. solanacearum* colonies. The typical *R. solanacearum* colonies were streaked on new CPG medium plates containing TTC and incubated at 28°C for 48 h. These colonies were further inoculated into conical flasks containing CPG liquid medium and grown overnight at 28°C with shaking at 200 rpm. The freshly prepared bacterial suspension culture was expandingly propagated for inoculation assay or genome DNA extraction.

### Peanut Planting and Inoculation

Peanut seeds were soaked in tap water for 4 h and then placed in glass culture dishes with wet filter paper. They were grown at 28 ± 1°C in a greenhouse with cycles of 16 h light/8 h night. After 2 days, germinated seeds were transferred into pots containing soil mix. Two weeks later, the seedlings with seven-to-eight full-grown leaves were used for inoculation experiments. The freshly prepared 20 μl bacterial suspension was added into a conical flask with CPG liquid medium and cultivated overnight to an OD_600 nm_ of approximately 1.0. Then, the cultured bacteria were centrifuged at 4,000 rpm for 10–15 min, and the pellet was re-suspended in water to OD_600 nm_ of 0.1. Two diagonal leaflets from the inverted third and fourth leaves were perpendicularly cut away (one-third of the leaflets was cut) by sterile scissors dipped the bacterial suspension. The control was inoculated with sterile water. The disease score of each plant was recorded every day after inoculation. The bacterial wilt severity of infected plants was divided into five grades: grade 1–few leaves wilt; grade 2–no more than 1/3 leaves wilt; grade 3–all leaves except the tip are wilt; grade 4–the whole plant wilts and dies. The disease index (DI) is calculated by the following formula: DI = 100Σn*i*/4N, where, *i* is the disease score of the plants; n is the number of plants showing the disease score of *i*; and N is the total number of the plants inoculated. The evaluation criteria of bacterial wilt resistance are divided into four levels: resistance (R), moderate resistance (MR), moderate susceptibility (MS), and susceptibility (S). The corresponding DIs were 0 ≤ DI < 25, 25 ≤ DI < 50, 50 ≤ DI < 75, and 75 ≤ DI < 100, respectively. A total of 72 peanut plants were inoculated for each strain. Three biological replicates were set with 24 plants per replicate. To calculate the survival ratio of the infected peanut plants, the disease grade was transformed into binary data according to the following criteria: a disease grade lower than 2 was defined as “0,” while a disease grade equal to or higher than 2 was defined as “1” for each specific time point (Remigi et al., 2011).

### Extraction of the Genomic DNA, Sequencing and Assembly

The genomic DNA of *R. solanacearum* strain PeaFJ1 was extracted using the HiPure Bacterial DNA Kit (Magen Bio, Guangzhou, China) according to the manufacturer’s protocols. The quality of the extracted DNA was evaluated using Qubit 2.0 Flurometer (Life Technologies, Carlsbad, CA, United States) and NanoDrop (Thermo Fisher Scientific, Wilmington, DE, United States). The qualified genomic DNA were used for complete genome sequencing through the PacBio long-read sequencer. The genomic DNA was fragmented and end-repaired to construct SMRTbell libraries (fragment sizes of > 10 Kb were selected by Blue Pippin system) according to the manufacturer’s specification (Pacific Biosciences, Menlo Park, CA, United States). The library quality was determined by Qubit 2.0 Flurometer (Life Technologies, Carlsbad, CA, United States) and its average fragment size was estimated at the Bioanalyzer 2100 (Agilent Technologies, Santa Clara, CA, United States). The single molecule real time (SMRT) sequencing was accomplished on the PacBio Sequel system (Pacific Biosciences, Menlo Park, CA, United States) according to standard protocols. The *de novo* assembly of the PacBio long-reads was performed using the program Falcon (version 0.3.0) with default parameters.

### Functional Annotation

The program Prokka (version 1.11), combined with National Center for Biotechnology Information (NCBI) prokaryotic genome annotation pipeline, was used to predict the open reading frames (ORFs) (Seemann, 2014; Tatusova et al., 2016). The predicted genes of *R. solanacearum* strain PeaFJ1 were annotated by BLASTN (*E*-value < 1e-5) using NCBI non-redundant protein (Nr) database, Swissport, Cluster of Orthologous Groups of proteins (COG), Kyoto Encyclopedia of Genes and Genomes (KEGG), and Gene Ontology (GO) databases based on sequence homology. The rRNAs, tRNA, and sRNA were identified using the program rRNAmmer (version 1.2), tRNAscan (version 1.3.1), and cmscan (version 1.1.2), respectively (Lagesen et al., 2007; Nawrocki and Eddy, 2013; Lowe and Chan, 2016). Genomic islands (GIs) were analyzed using the Island Viewer online tool (version 4.0)^2^ (Bertelli et al., 2017). Prophages were identified using the program Phage Finder (version 2.0) (Fouts, 2006). Protein family annotation was performed with Pfam_Scan (version 1.6) based on the Pfam database (version 32.0) (Finn et al., 2014). Blastp and Blastn with default parameters were used to compare annotations to the Pathogen Host Interactions (PHI) and Virulence Factors of Pathogenic Bacteria (VFDB) databases. Two-component systems (TCSs) were predicted based on their structure characteristics (Cheung and Hendrickson, 2010). Type III effectors were identified according to the Ralsto T3E database (Sabbagh et al., 2019) and the gene functional annotation. All analyses were carried out using the default parameters.

### Comparative Genomic Analysis

MUMmer software was used to compare the target genome with the reference genome to determine the large range of collinearity between genomes (Kurtz et al., 2004). Then, SyRI was used to make comparison between regions, confirm local location arrangement relationship, and find translocation and inversion regions (Goel et al., 2019). The *R. solanacearum* PeaFJ1 and HA4-1 genome alignments were carried out in an all-against-all comparison using the MUMmer 3 package (version 3.2.2) with default parameters (Kurtz et al., 2004). Orthologous gene clusters in the genomes of PeaFJ1 and other strains were identified consecutively through combining the program DIAMOND and the program OrthoMCL (version 2.0) (Silva-Pereira et al., 2019). Then, all the putative proteins of the PeaFJ1 and core orthologs were aligned using BLASTP (Hirsh and Fraser, 2001). In addition, the strain-specific genes of PeaFJ1 compared with HA4-1 were analyzed for screening the candidates for the difference pathogenicity to peanut. GO and KEGG enrichment analyses were performed using the functional annotation tool DAVID (version 6.8) with default parameters (Dennis et al., 2003; Sherman et al., 2007).

### Statistical Analysis

Statistical analyses and graphs were generated by using the GraphPad Prism 8.0 software. The *p*-values less than 0.05 indicate significant differences among the survival ratio of HA4-1 and PeaFJ1.

## Results and Discussion

### PeaFJ1 Strain Shows More Aggressive to Most Peanut Varieties

**FIGURE 1:**
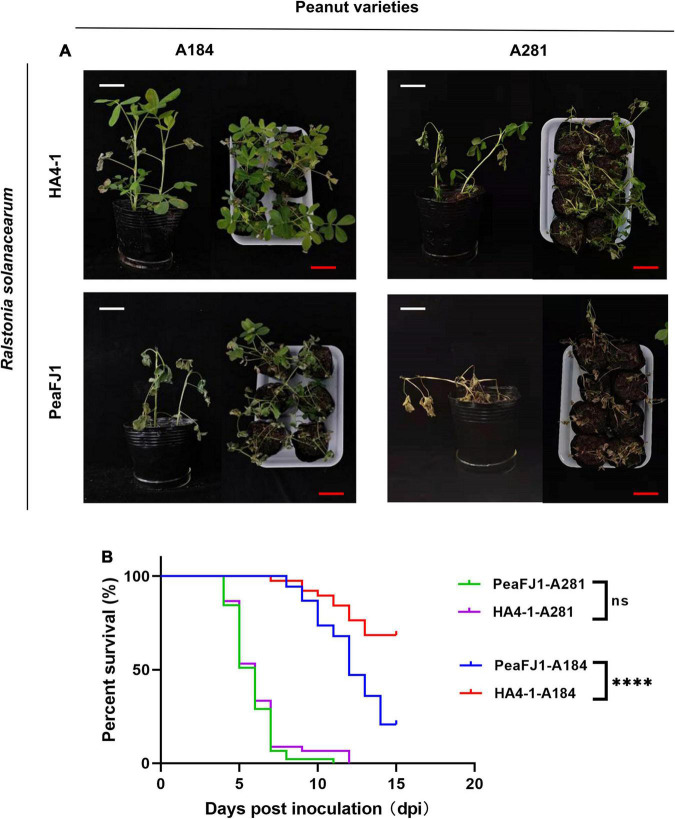
*R. solanacearum* PeaFJ1 shows different virulent profiles compared with HA4-1 in peanut varieties. Bacterial wilt symptoms **(A)** and survival ratio **(B)** of the peanut varieties A184 and A281 infected with *R. solanacearum* PeaFJ1 and HA4-1, respectively. **(A)** Photographs from representative plants were taken at 10 days post- inoculation. White scale bars = 5 cm, red scale bars = 10 cm. **(B)** The percentage of surviving peanuts was recorded for 15 days. The data used for the survival ratio were collected from three independent experiments. Gehan–Breslow–Wilcoxon test *p*-values are < 0.0001 and 0.6471 in A184 and A281, respectively. ****indicates *p* < 0.0001, (ns) means no significantly different.

To screen bacterial wilt-resistant and -susceptible sources, we evaluated 303 cultivated peanut varieties for disease susceptibility to two *R. solanacearum* strains PeaFJ1 and HA4-1. We found that PeaFJ1 was more aggressive to most varieties than HA4-1 according to the wilting symptoms and DI. To confirm this result, we randomly selected 113 varieties to repeat the inoculation assay. Of the 113 varieties, 85 were susceptible to PeaFJ1 and 62 were susceptible to HA4-1. The susceptibility rates were 75% (85/113) and 55% (62/113), respectively (Supplementary Figure 1A). In addition, in the DI of most varieties, inoculated PeaFJ1 was higher than that of inoculated HA4-1. For some peanut varieties, the pathogenicity of the two strains was significantly different (Supplementary Figure 1B). For example, PeaFJ1 showed reproducible and robust hypervirulence phenotypes, while HA4-1 showed hypovirulent phenotypes, in peanut variety A184. However, for a few varieties, the DI was equal or higher after inoculation with HA4-1 than with PeaFJ1. For example, HA4-1 could cause strong disease symptoms in another peanut variety A281, which indicates that it retains its intrinsic pathogenicity as a pathogen (Figure 1). These results indicated that PeaFJ1 and HA4-1 had different virulence profiles, and PeaFJ1 was more virulent to most peanut varieties than HA4-1. The hypovirulence of HA4-1 to certain peanut varieties is probably owed to host specificity at genotype levels, which is closely related to the type III effectors, since T3Es always narrow the host range when certain effectors are specifically recognized as avirulence factors by the host (Jones and Dangl, 2006; Chiesa et al., 2019; Cho et al., 2019; Tan et al., 2019). These data suggest that HA4-1 may contain an avirulence T3E(s) that induces bacterial wilt disease resistance in A184. Further phylogenetic trees analysis based on the similarity of endoglucanase gene sequence revealed that PeaFJ1 and HA4-1 were close phylogenetically and both belonged to phylotype I sequevar 14 (Supplementary Figure 2A), indicating that the genome differences between the two strains will be relatively small. This is a great advantage for us to use comparative genomic analysis to find the key genes that determine PeaFJ1 stronger pathogenicity in most peanut varieties. In addition, considering the A184-specific avirulence phenotype of HA4-1, we can also screen some candidate avirulence T3Es for further analysis by screening the absent T3Es in the PeaFJ1 genome.

### Sequencing, Assembly and Overview of the PeaFJ1 Genome

**FIGURE 2:**
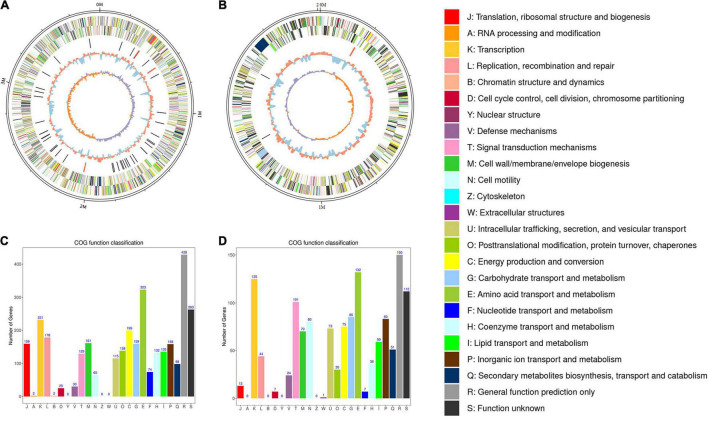
Circos plot of the genome of *R. solanacearum* strain PeaFJ1. The genome of strain PeaFJ1 consists of a chromosome contig with a full length of 3,800,378 bp **(A)** and a megaplasmid contig with a full length of 2,008,534 bp **(B)**. From the outer circle to the inner circle, it represents the length of chromosomes, CDS, tRNAs, GC content, and GC skew curve, respectively. **(C,D)** The distribution of genes with COG functional categories in the chromosome **(C)** and the megaplasmid **(D)** of PeaFJ1. Different colors represent different COG functional classifications that were explained in the right side.

**FIGURE 3:**
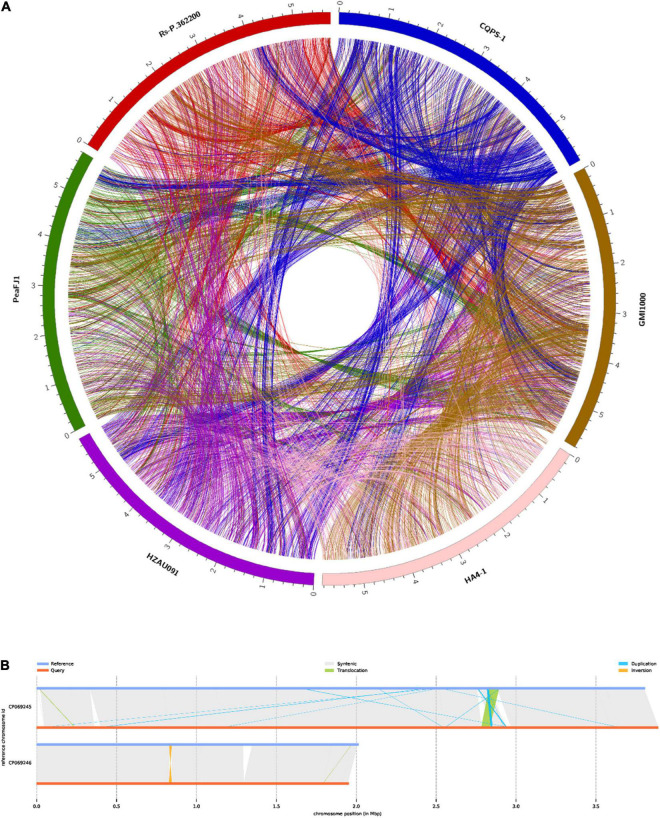
Comparison of *R. solanacearum* PeaFJ1 with other representative sequenced *R. solanacearum* strains. **(A)** Synteny blocks identified in PeaFJ1 across HA4-1, Rs-P.362200, HZAU091, GMI1000, and CQPS-1. **(B)** Illustration of structural variation types between PeaFJ1 and HA4-1. Reference is HA4-1, and query is PeaFJ1.

The genome of *R. solanacearum* PeaFJ1 was sequenced using the PacBio Sequel platform. As a result, a total of 1.4 Gb polymerase reads from a 20 kb library were generated by SMRT sequencing. After removing adapters and low-quality or ambiguous reads, we obtained 1.4 GB (∼241 ×) subreads for complete genome assembly of PeaFJ1. The PeaFJ1 genome consists of a circle chromosome contig of 3,800,378 bp with 66.78% GC content and a circle megaplasmid contig of 2,008,534 bp with 66.97% GC content (Figures 2A,B and Table 1). No small plasmid was found in PeaFJ1. The full length of PeaFJ1 chromosome is relatively smaller than that of HA4-1 (3,800,378 bp vs. 3,890,347 bp) while the megaplasmid is larger than that of HA4-1 (2,008,534 bp vs. 1,947,245 bp). As a whole, the genome size of PeaFJ1 is slightly smaller than that of HA4-1 (5,808,912 bp vs. 5,837,592 bp) (Tan et al., 2019). In order to compare the genome of PeaFJ1 with other sequenced phylotype I *R. solanacearum* genomes, the synteny blocks between PeaFJ1 and other representative sequenced *R. solanacearum* strains such as HA4-1, Rs-P.362200, HZAU091, GMI1000, and CQPS-1 were identified using the program C-Sibelia (Minkin et al., 2013; Figure 3A). The results revealed that the PeaFJ1 genome situates a higher syntenic relationship with HA4-1, Rs-P.362200, and HZAU091. The number of collinear blocks are 20, 26, and 24, respectively. The proportion of the total base length of collinear blocks accounts for more than 97% of the PeaFJ1 genome, whereas 80 and 82 collinear blocks were identified in PeaFJ1 when compared with GMI1000 and CQPS-1. The full length of collinear blocks accounts for 91.9 and 93.34% in the genome of PeaFJ1, respectively. It suggests that the genome of PeaFJ1 is more similar with HA4-1, Rs-P.362200, and HZAU091. *R. solanacearum* strains HA4-1 and HZAU091 both belonged to phylotype I and sequevar 14M and isolated from peanut and potato, respectively (Wang et al., 2017; Tan et al., 2019). The *R. solanacearum* strain Rs-P.362200 was isolated from peanut and belonged to phylotype I (Chen et al., 2021). The GMI1000 is most widely used as a standard strain, isolated from the tomato and belonged to phylotype I, sequevar 18, while the CQPS-1 is isolated from the tobacco (Liu et al., 2017). The result of synteny analysis indicates that the genomic similarity of *R. solanacearum* strains could be preliminarily estimated by the sequevar identification or their host. Structural variation (SV) is the insertion, deletion, inversion, and translocation of the long sequence fragments whose length is more than 50 bp. Here, we detected 5 translocated regions, 15 duplicated regions, and 2 inverted regions between the genome of PeaFJ1 and HA4-1 (Figure 3B). In addition, four inverted duplicated regions and one inverted translocated region were also identified. The difference in virulence between PeaFJ1 and HA4-1 may be closely related to these variations.

**TABLE 1:** General features of the *R. solanacearum* PeaFJ1 genome.

Features	Values
Genome size (bp)	5,808,912
Chromosome (bp)	3,800,378
Megaplasmid (bp)	2,008,534
G + C content (%)	66.88
ORFs	5,098
Predicted genes	4,869
Pseudogenes	155
rRNA	12
tRNA	59
sRNA	8
Genomic islands	21
Prophages	2

### Functional Annotation of the PeaFJ1 Genome

The general characteristics of the PeaFJ1 genome are listed in Table 1. There are 5,098 ORFs identified (3,502 in the chromosome and 1,596 in the megaplasmid) in the genome of *R. solanacearum* PeaFJ1. By searching several databases, 3,342 and 1,527 coding genes were predicted in the chromosome and the megaplasmid, respectively. Of all the 4,869 genes, 4,865 (99.92%), 2,886 (59.27%), 3,686 (75.7%), and 2,579 (52.97%) genes were annotated according to NCBI Nr, SwissProt, COG, and KEGG databases, respectively (Supplementary Table 1). In addition, 155 pseudogenes were identified in the whole genome. Functional annotation successfully classified 2,640 chromosome genes into 22 COG categories and 1,647 megaplasmid genes into 21 COG categories (Figures 2C,D). Although more genes are in the chromosome, there are more genes related to cell motility in the megaplasmid. The PeaFJ1 genome contains 12 rRNAs, 59 tRNAs, and 8 sRNAs (Table 1). The full length of ncRNAs is 23,714 bp, accounting for 0.75% of the PeaFJ1 whole genome sequence. Virulence factor is an important basis of bacterial virulence, which plays a major role in the pathogenesis of the pathogen. The biological function analysis of virulence factor has become the primary task of the pathogenic mechanism study. There are 351 genes in PeaFJ1 having homologs in the VFDB database (Supplementary Table 2), which is a pathogen virulence factor database.^3^ These genes are highly homologous with those genes in other pathogens that have been shown to contribute to their virulence. There are 1,085 genes having homologs in the PHI database (Supplementary Table 2), which collects sequences of experimentally validated pathogenic and effector genes from the literature.^4^ The homologous genes of these genes have been experimentally confirmed to cause certain diseases in certain hosts and to interact with certain genes in the host. These predictions could help us quickly identify the key genes that cause PeaFJ1 to be more virulent to most peanut varieties.

### Genomic Islands and Prophage Elements

**FIGURE 4:**
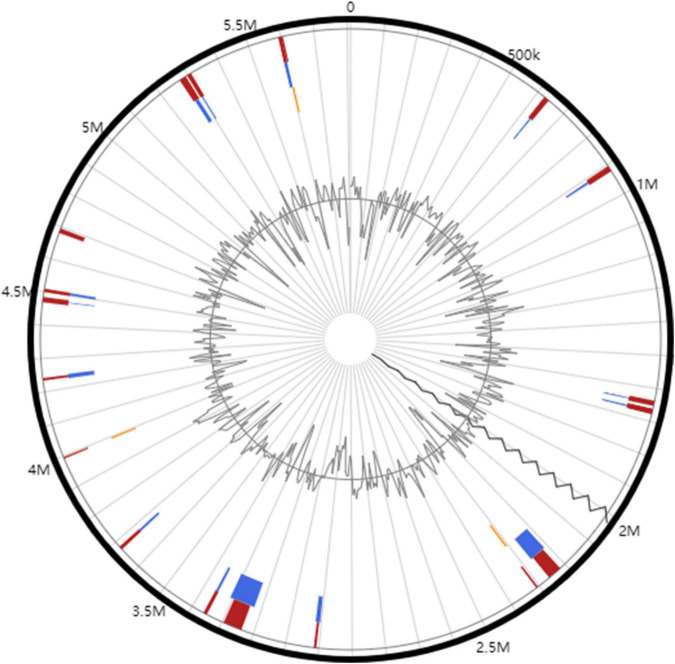
Circular plots of genomic islands identified in *R. solanacearum* PeaFJ1 genome. The orange- and blue-colored shapes determine the predicted genomic islands as identified by SIGI-HMM (orange) and IslandPath-DIMOB (blue), and red shows the integrated genomic island search results.

Horizontal gene transfer (HGT) can enhance the adaptability of bacteria to the environments. Genomic islands and prophages are the most important mobile elements in HGT (Fouts, 2006). The coding regions of genomic islands usually contain large numbers of virulence gene clusters, which encode the virulence factors in many pathogenic bacteria (Tatusova et al., 2016). In total, 21 genomic islands (GIs) were predicted in the whole genome (16 located in the chromosome and 5 located in the megaplasmid) of PeaFJ1 (Figure 4 and Supplementary Table 3). The total length of the GIs is 475,829 bp, which accounts for 8.2% of the PeaFJ1 genome. There are three type III effectors located in the GIs, which are RipP1, RipAX2, and RipG2. There are five GIs longer than 35 kb, and each containing more than 30 genes. The prophage sequences may confer some bacteria antibiotic resistance, improve bacterial adaptability to the environments, strengthen bacterial adhesion, or cause the bacteria to be more pathogenic (Bertelli et al., 2017). There are two prophages predicted in the chromosome, whose sizes are 13,278 and 32,359 bp (Supplementary Table 4). There are 15 and 42 genes in the two prophage regions, respectively. The GC content of the prophages is lower than that of the chromosome (64.34%). The bacteriophages best matched to the two prophages are NC_004821 and NC_009382. One of the prophage regions partially overlaps with the GIs. HGT plays an important role in *R. solanacearum* genetic and pathogenic diversity by contributing to the rapid acquisition of novel adaptive functions and pathogenic factors and is thus crucial for the adaptation and the emergence of pathogenic variants. Therefore, the HGT-related genes of PeaFJ1 are worthy of attention in the future study.

### Specific Genes Analysis of PeaFJ1

**FIGURE 5:**
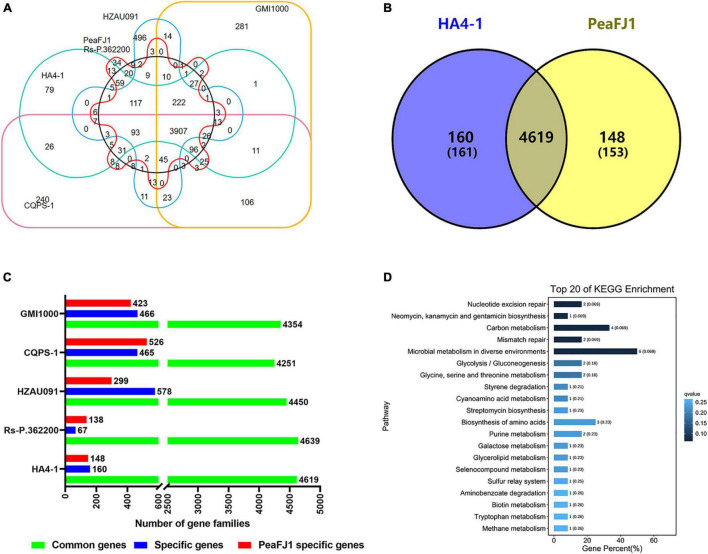
Comparison of orthologs of strain PeaFJ1 with other *R. solanacearum* strains. **(A)** A big Venn diagram of orthologs for PeaFJ1, HA4-1, Rs-P.362200, HZAU091, GMI1000, and CQPS-1. **(B)** Statistical analysis of common/specific orthologs between PeaFJ1 and the other five strains. The *x*-axis indicates the number of gene families; the *y*-axis means the different *R. solanacearum* strains. Green bar, blue bar, and red bar mean common gene families, specific gene families, and PeaFJ1- specific gene families when PeaFJ1 is compared with the other five strains, respectively. **(C)** A Venn diagram of the common/specific genes between PeaFJ1 and HA4-1. **(D)** Top 20 enriched KEGG pathways of strain-specific genes in PeaFJ1 whole genome. The *q*-value results from the *p*-value *via* multi-test correction. The ranges of *q*-value are from 0 to 1, and a higher enrichment is achieved when the *q*-value reaches to 0.

The genomic comparison of the PeaFJ1 with the other five sequenced *R. solanacearum* strains was carried out using the genomic protein sequences, and the unique gene families of PeaFJ1 were identified (Figure 5A). Gene family analysis showed that there were 4,869 gene clusters in the genome of the PeaFJ1 strain, which could be classified into 4,744 gene families, among which 34 gene families were unique to PeaFJ1 (Supplementary Table 5). By analyzing the specific gene families of PeaFJ1 compared with other strains, there were 4,619, 4,639, and 4,450 common gene families, 148, 138, and 299 unique gene families in PeaFJ1 compared with strains HA4-1, Rs-P.362200, and HZAU091, respectively (Figure 5B). While compared with CQPS-1 and GMI1000, there were 4,251 and 4,354 common gene families and 526 and 423 specific gene families in PeaFJ1. It indicated that PeaFJ1 was more closely related to the three strains HA4-1, Rs-P.362200, and HZAU091, consistent with the ANI (average nucleotide identity) analysis results (Supplementary Figure 2B).

Because *R. solanacearum* PeaFJ1 is more pathogenic than HA4-1, the specific genes in PeaFJ1 compared with HA4-1 could be the determinants. By comparative genomic analysis, we identified 153 (in 148 gene families) and 161 (in 160 gene families) specific genes in PeaFJ1 and HA4-1 genomes, respectively (Figure 5C and Supplementary Table 6). To further reveal the gene ontology and functional classification of these PeaFJ1-specific genes, we performed the GO and KEGG enrichment analysis. Among the strain-specific genes of PeaFJ1, 49 (59.04%), 29 (34.94%), and 5 (6.02%) GO terms were enriched in the three categories of biological process, molecular function, and cellular component, respectively (Supplementary Table 7). KEGG enrichment analysis demonstrated that PeaFJ1-specific genes are mainly enriched in microbial metabolism in diverse environments (ko01120), carbon metabolism (ko01200), and the biosynthesis of amino acids (ko01230) (Figure 5D). Further analysis of these strain- specific genes showed that most of the specific genes (114 genes) are located in six gene clusters (Supplementary Table 6). The largest gene cluster consists of 38 genes (from JNO62_20500 to JNO62_20690). Four of the gene clusters almost overlap with the GIs. There are a total of 58 PeaFJ1-specific genes located on the GIs, accounting for 38% of the specific genes. One of the gene clusters consisting of 14 genes (from JNO62_05980 to JNO62_06045) is located on the prophage region. The above data suggested that many specific genes of PeaFJ1 are located on the mobile elements and related to HGT. The genes matched to the VFDB database and PHI database are important for the pathogenicity and host interaction for the pathogens. Here, we found that there were 7 and 20 specific genes, respectively, which were homologous with genes in the two databases (Supplementary Table 2).

#### Swimming Motility-Related Genes

*R. solanacearum* has evolved with different movement strategies to reach different plant tissues and get inside the vascular system, of which swimming motility is an individual cell movement produced in aqueous environments and powered by rotating flagella (Corral et al., 2020). In *R. solanacearum*, swimming motility is mediated by one to four polar flagella (Tans-Kersten et al., 2001). The flagellum comprises three functional parts: a thin helical flagellar filament that acts as a propeller, a reversible rotary molecular motor embedded on the envelope, and a hook that acts as a universal connection joint between the motor and the flagellar filament (Macnab, 2003). Among the PeaFJ1 strain-specific genes, one gene was functionally annotated to be the *fliK* gene (JNO62_02405), which encodes a protein with a flagellar hook length control motif (Supplementary Table 6). The bacterial flagellum is a complex organelle of the cell. The *fliK* gene is required for flagellar filament assembly and function (Evans et al., 2014). A mutant of the *fliK* gene in *Bacillus thuringiensis* completely failed to produce detectable flagellar filaments, and its biofilm formation is highly compromised as well. Both flagellar assembly and swimming motility are restored by the functional complementation of the mutant strains by the *fliK* ORF (Attieh et al., 2020). These results confirm the essential function of the FliK protein in the flagellar assembly, motility, and biofilm formation in *B. thuringiensis*. Compared with HA4-1, the predicted *fliK* gene is unique in PeaFJ1, indicating that it is probably responsible for the more aggressiveness of PeaFJ1 strain. In the future, systematic experiments including mutant construction were needed to verify the function of the *fliK* gene of *R. solanacearum* both *in vitro* and *in vivo* and especially in peanuts.

#### LysR-Type Transcriptional Regulators

The LysR*-*type transcriptional regulators represent the most abundant type of transcriptional regulators in the prokaryotic kingdom. By affecting the efficiency of transcription initiation, the LysR-type transcriptional regulators regulate a diverse set of genes, including those involved in virulence, metabolism, quorum sensing, and motility (Hernández-Lucas et al., 2008; Maddocks and Oyston, 2008). To date, many LysR-type transcriptional regulators have been identified to be important for the virulence of many pathogenic bacteria (Huang et al., 1998; Habdas et al., 2010; Rashid et al., 2016). In the *R. solanacearum*, a quorum-sensing-dependent LysR-type transcriptional regulator PhcA has been well characterized as a global regulator that controls the expression of diverse virulence-related genes, including those involved in plant cell wall degradation, motility, synthesis of EPS, and the T3SS (Brumbley et al., 1993; Huang et al., 1995; Genin et al., 2005). Among the PeaFJ1 strain-specific genes, three genes were functionally annotated to be LysR-type transcriptional regulators (JNO62_02440, JNO62_02445, and JNO62_20640) (Supplementary Table 6). Relative to HA4-1, the strong virulence of PeaFJ1 may be due to the presence of these three genes.

#### Adhesion- and Invasion-Related Genes

Lipoproteins, as an important part of outer membrane proteins that are widely distributed in Gram-negative bacteria, have been shown to perform a variety of roles in bacterial physiological and pathogenic processes (Kovacs-Simon et al., 2011; Sutcliffe et al., 2012). VacJ protein is a recently discovered outer membrane lipoprotein that relates to virulence in several pathogens. It plays an essential role in maintaining outer membrane integrity, stress tolerance, biofilm formation, adherence to, and invasion in host cells related to the pathogen (Zhao et al., 2017). Adherence to the host cell surface is an essential process for bacterial colonization and cellular invasion, which contribute to the breaching of the cell barrier, staying inside the host and ultimately leading to systemic disease (Vahle et al., 1997). Adhesion and invasion of host cells are also considered as important factors for pathogenesis in *R. solanacearum*. Among the PeaFJ1 strain-specific genes, one gene was functionally annotated to be the *VacJ* gene (JNO62_18020) (Supplementary Table 6). As a plant bacterial pathogen that colonizes in the vascular system, *R. solanacearum* infecting the host also depends on the adhesion and invasion of host cells. Thus, the *VacJ* gene in the PeaFJ1 but not in HA4-1 may be responsible for their different pathogenicity to peanuts. The definite function of *VacJ* gene in PeaFJ1 is to be studied by constructing the mutant strain in the following research.

#### Two-Component Systems-Related Genes

Bacteria alter their gene expression in response to environment changes through a variety of mechanisms including signal transduction systems. These signal transduction systems use kinase with extracellular or periplasmic sensing domains to transfer phosphate groups to DNA-binding molecules and consequently induce the gene expression change. Bacterial signal-transduction systems often involve only two proteins (a sensing protein and a transcription factor), and are thus called TCSs (Stock et al., 2000; Groisman, 2016). In the PeaFJ1 genome, there are 32 TCSs (19 located in the chromosome and 13 located in the megaplasmid) (Supplementary Figure 3 and Supplementary Table 8). Among the PeaFJ1 strain-specific genes, four genes were functionally annotated to be TCS-related genes. The four genes are JNO62_20595, JNO62_20600, JNO62_20655, and JNO62_20660 (Supplementary Tables 6, 8), among which, JNO62_20595 and JNO62_20655 were predicted to encode sensor kinases while JNO62_20600 and JNO62_20660 were predicted to encode response regulators. The four genes make up two TCSs located in the megaplasmid. So far, no studies on TCS have been reported in *R. solanacearum.* TCSs are known to play important roles in bacterial motility and chemotaxis, physiological responses to osmotic changes, biofilm formation, and the regulation of virulence in many bacteria (Stock et al., 2000; Freeman et al., 2013; Groisman, 2016). Whether these two PeaFJ1-specific TCSs are responsible for the different virulence between PeaFJ1 and HA4-1 needs further research.

#### Type III Effector Genes

The secretory systems and their secreted components are always important for the virulence of pathogens, among which, the T3SS and its effectors especially attracted more attention of the researchers. T3Es are secreted by the “molecular syringe-like” T3SS and translocated into the cell to play versatile functions when interacting with the host. Many studies have showed that *R. solanacearum* effectors can regulate the host metabolism, suppress plant defense responses, or avoid bacterial recognition through various molecular mechanisms. For example, the effector RipG belonging to the GAxALA (GALA) effector protein family can inhibit the ubiquitination signal in the host and then promote the infection response of *R. solanacearum* (Angot et al., 2006; Remigi et al., 2011). RipAY can be selectively activated by thioredoxin to degrade glutathione in the host, providing a suitable environment for *R. solanacearum* infection (Wei et al., 2017; Sang et al., 2018). RipN acts as a nudix hydrolase, alters the nicotinamide adenine dinucleotide (NADH)/nicotinamide adenine dinucleotide (NAD^+^) ratio of the plant, and contributes to virulence by suppression of PAMP-triggered immunity (PTI) of the host (Sun et al., 2018). RipAB inhibits the calcium-signaling pathway, which in turn decreases the accumulation of reactive oxygen species mediated by Pathogen-associated molecular patterns (PAMPs), thus making the transgenic potatoes of *RipAB* more susceptible to *R. solanacearum* (Zheng et al., 2018). RipV2 has the activity of E3 ubiquitin ligase, which can cause cell necrosis in tobacco leaves and inhibit the PTI immune defense response in plants (Cheng et al., 2021). RipI interacts with plant glutamate decarboxylases to alter plant metabolism and support bacterial growth (Xian et al., 2020). On the other hand, *R. solanacearum* T3Es can be perceived by the plant and activate the defense response in the host. RipP2, an effector protein of YopJ family, has acetyltransferase activity. It can acetylate the WRKY domain of nucleotide-binding leucine-rich repeat receptor (NLR)-resistant proteins and stimulate immune resistance to *Arabidopsis thaliana*. RipP1, which also belongs to the YopJ family with acetyltransferase activity, can induce an immune response in petunias (Poueymiro et al., 2009). RipB induced bacterial wilt resistance in tobacco by the recognition of Roq1 (Nakano and Mukaihara, 2019). RipAX2 elicited immune responses in wild and cultivated eggplants, which were dependent on the Zn-finger domain in wild eggplants but not in cultivated eggplants (Nahar et al., 2014; Morel et al., 2018). Although every *R. solanacearum* has more than 50 effectors, different strains have different repertoire of effectors. In order to reveal the effector repertoire of *R. solanacearum* PeaFJ1, we identified the effectors according to the Ralsto T3E database (Sabbagh et al., 2019) and the gene functional annotation. As a result, 79 type III effectors were identified, among which 11 are pseudogenes (Supplementary Table 9).

In order to quickly identify the key effectors related to pathogenicity of pathogens from numerous effectors, a lot of strategies have been performed by researchers. Some T3Es with important functions are selected out largely depending on the comparative analysis of the effectors. For instance, the recently reported *R. solanacearum* avirulence effectors RipJ and RipAZ1 are identified by the comparative genomic analysis of two strains with different virulence against *S. pimpinellifolium* and *S. americanum*, respectively (Moon et al., 2021; Pandey and Moon, 2021). Similarly, by comparing all the effectors of PeaFJ1 and HA4-1, we found that most of the effectors are exactly identical with the counterparts of *R. solanacearum* HA4-1. Only a few effectors are discrepant in the two strains (Supplementary Table 9). RipS4 is present in both stains, whereas it is prematurely terminated due to a base pair deletion in HA4-1. As the C-terminal of a protein always plays an important role in its function, the difference of RipS4 may be responsible for the difference of virulence between the two strains. A single copy of *RipBR* gene was identified in the megaplasmid of PeaFJ1, while two copies of *RipBR* gene were located in the megaplasmid and small plasmid of HA4-1, respectively, although the latter was inserted by a transposon (Tan et al., 2019). There are three copies of *RipBS* gene existing in the genome and small plasmid of HA4-1, while no *RipBS* gene was found in the genome of PeaFJ1. In addition, RS_T3E_Hyp6 is absent in PeaFJ1, while it is present in HA4-1. On the contrary, RipBB is present in PeaFJ1 but not HA4-1 (Supplementary Table 9). According to the general function of effectors, RipBB may be responsible for the higher pathogenicity of PeaFJ1 against peanuts. That is to say, RipS4 and RipBB are virulence effectors. The hypovirulence of HA4-1 to certain peanut varieties may be due to the presence of RipBS and RS_T3E_Hyp6. That is to say, RipBS and RS_T3E_Hyp6 may be avirulence effectors. The specific roles of these four effectors acting on peanuts need to be explored by systematic experiments in the following study. There is a minor difference in RipG5 and RipH1 genes between PeaFJ1 and HA4-1. However, it cannot be ruled out that one or two amino acid differences may cause differences in effector functions. The above-mentioned T3Es will be an important direction for the mechanism research of *R. solanacearum*–peanut interaction, so a series of experiments are required to explore the particular roles of these effectors in the future.

## Conclusion

In this study, we completed a high-quality sequencing and analysis of a strong virulent *R. solanacearum* strain PeaFJ1 isolated from peanut. In view of the pathogen research of peanut bacterial wilt still being scarce, the results of the genome analysis and the pathogenicity of PeaFJ1 to various peanut varieties will provide strong support for the study of the interaction between *R. solanacearum* and peanut. Through a detailed comparative analysis of the genomes between PeaFJ1 and another *R. solanacearum* strain HA4-1 from peanut, we screened out the strain-specific genes of PeaFJ1, which may be the key factors causing different virulence profiles between the two strains. It is the first time to explore the key genes of *R. solanacearum* pathogenicity by doing a comparative genomic analysis of two different virulence strains from peanut. Our study enriches the information of the *R. solanacearum* species complex genomes and contributes to gain a deeper insight into the pathogenic factors of *R. solanacearum* against peanut as well. These results will greatly promote the study of interaction mechanism between peanut and *R. solanacearum* and will also be beneficial to the development of peanut bacterial wilt control agents.

## Data Availability Statement

The datasets presented in this study can be found in online repositories. The names of the repository/repositories and accession number(s) can be found in the article/Supplementary Material.

## Author Contributions

XT and YY designed the research, analyzed the data, and wrote the manuscript with contributions of all the authors. XD and TC performed the experiments and analyzed the data. DY, YW, and YY provided technical assistance to XT. YZ and HC supervised the experiments. XW supervised and complemented the writing. All authors contributed to the article and approved the submitted version.

## Conflict of Interest

The authors declare that the research was conducted in the absence of any commercial or financial relationships that could be construed as a potential conflict of interest.

## Publisher’s Note

All claims expressed in this article are solely those of the authors and do not necessarily represent those of their affiliated organizations, or those of the publisher, the editors and the reviewers. Any product that may be evaluated in this article, or claim that may be made by its manufacturer, is not guaranteed or endorsed by the publisher.

## Footnotes

1
https://www.fao.org/faostat/en/#home

2
http://www.pathogenomics.sfu.ca/islandviewer/upload/

3
http://www.mgc.ac.cn/VFs/main.htm

4
http://www.phi-base.org/

## Supplementary Material

Supplementary Figure 1 PeaFJ1 have different virulence profiles with HA4-1 on different peanut varieties. **(A)** The heatmap of disease grade of 113 peanut varieties after 10 days inoculated with *R. solanacearum* HA4-1 and PeaFJ1, respectively. The column is *R. solanacearum* HA4-1 and PeaFJ1, and the row is the different peanut varieties. R, Resistance, 0 ≤ DI < 25; MR, Medium Resistance, 25 ≤ DI < 50; MS, Medium Susceptible, 50 ≤ DI < 75; S, Susceptible, 75 ≤ DI < 100. **(B)** The DI of 113 peanut varieties after 10 days inoculated with *R. solanacearum* HA4-1 and PeaFJ1, respectively. Significant differences were assessed by ANOVA and indicated by asterisks; single asterisk (*) indicates *P* < 0.05, and double asterisk (**) indicates *P* < 0.01. Values are means of three biological replicates, with error bars indicating the SD.

Supplementary Figure 2 Genetic relationship analysis of PeaFJ1 with other *R. solanacearum* strains. **(A)** Phylogenetic tree constructed with different *R. solanacearum* strains including HA4-1- and PeaFJ1-based *egl* gene sequences. The result confirms that HA4-1 and PeaFJ1 are closely phylogenetical and belong to phylotype I sequevar 14. HA4-1 and PeaFJ1 is indicated by a red box. Alignments were conducted in ClustalW, and the phylogenetic tree was constructed by the neighbor-joining algorithm in MEGA 6 software. Bootstrap values (1,000 replicates) are shown as percentages at the branch nodes. **(B)** The heatmap of ANI percentage identity for PeaFJ1 with other *R. solanacearum* strains. The number in the heatmap indicates different ANI percentage.

Supplementary Figure 3 Schematic diagram of two-component systems in *R. solanacearum* PeaFJ1. **(A)** The 19 TCSs in the chromosome. **(B)** The 13 TCSs in the megaplasmid. HisKA (blue): Phosphate receptor domain containing an H-box of conserved histidine sites capable of autophosphorylation. HATPase_c (red) is the intracellular catalytic domain of histidine kinase, responsible for the transfer of ATP phosphate groups to histidine. Response_reg (green): Response regulator (RR), response regulator protein. Black module: transmembrane helix. Numbers at both ends: gene ID.

Supplementary Table 1 Basic annotation of PeaFJ1 chromosome and megaplasmid.

Supplementary Table 2 PeaFJ1 genes annotated in VFDB and PHI database.

Supplementary Table 3 General features of genomic islands of PeaFJ1 genome.

Supplementary Table 4 General features of genomic prophages of PeaFJ1 genome.

Supplementary Table 5 The gene family of PeaFJ1 compared with other five sequenced strains.

Supplementary Table 6 *R. solanacearum* PeaFJ strain-specific genes compared with HA4-1.

Supplementary Table 7 GO enrichment analysis of PeaFJ1-specific genes compared with HA4-1.

Supplementary Table 8 TCS prediction results in *R. solanacearum* PeaFJ1.

Supplementary Table 9 Comparison analysis of type III effector genes of strain PeaFJ1 with strain HA4-1.
