# Supplementary figures and images for: Complete Genome Sequence Analysis of Ralstonia solanacearum Strain PeaFJ1 Provides Insights Into Its Strong Virulence in Peanut Plants
Source: Front Microbiol. 2022 Feb 23;13:830900. doi: 10.3389/fmicb.2022.830900 (PMC8904134; doi:10.3389/fmicb.2022.830900)

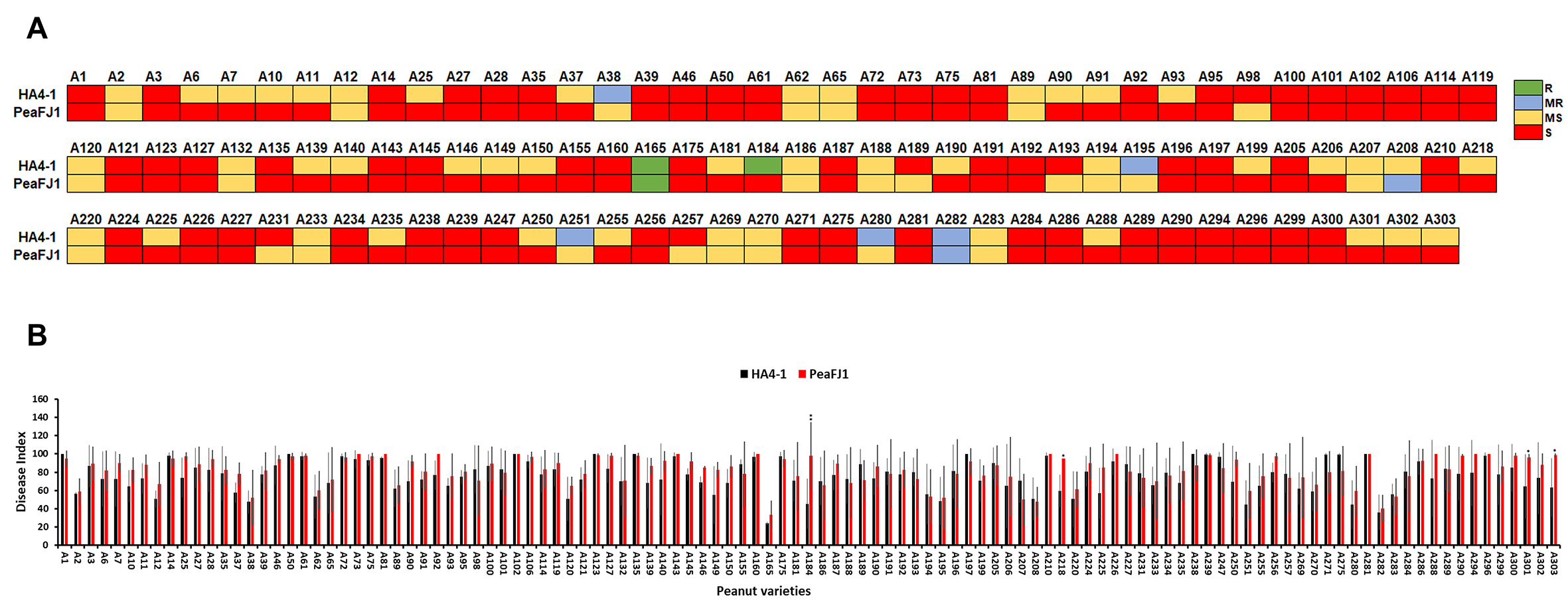

Supplement: Supplementary Figure 1 — PeaFJ1 have different virulence profiles with HA4-1 on different peanut varieties. (A) The heatmap of disease grade of 113 peanut varieties after 10 days inoculated with R. solanacearum HA4-1 and PeaFJ1, respectively. The column is R. solanacearum HA4-1 and PeaFJ1, and the row is the different peanut varieties. R, Resistance, 0 ≤ DI < 25; MR, Medium Resistance, 25 ≤ DI < 50; MS, Medium Susceptible, 50 ≤ DI < 75; S, Susceptible, 75 ≤ DI < 100. (B) The DI of 113 peanut varieties after 10 days inoculated with R. solanacearum HA4-1 and PeaFJ1, respectively. Significant differences were assessed by ANOVA and indicated by asterisks; single asterisk (*) indicates P < 0.05, and double asterisk (**) indicates P < 0.01. Values are means of three biological replicates, with error bars indicating the SD. [file Image_1.tif]

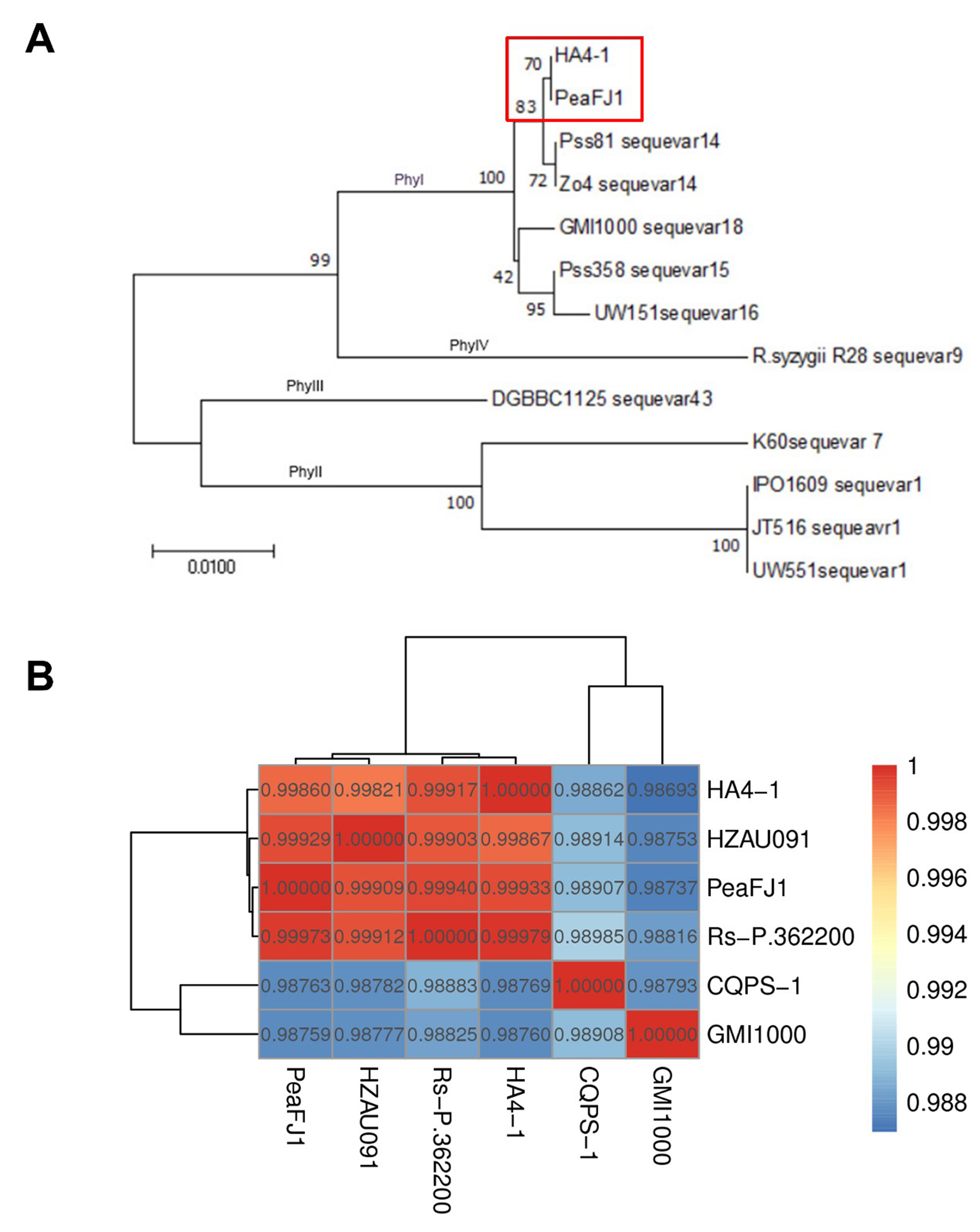

Supplement: Supplementary Figure 2 — Genetic relationship analysis of PeaFJ1 with other R. solanacearum strains. (A) Phylogenetic tree constructed with different R. solanacearum strains including HA4-1- and PeaFJ1-based egl gene sequences. The result confirms that HA4-1 and PeaFJ1 are closely phylogenetical and belong to phylotype I sequevar 14. HA4-1 and PeaFJ1 is indicated by a red box. Alignments were conducted in ClustalW, and the phylogenetic tree was constructed by the neighbor-joining algorithm in MEGA 6 software. Bootstrap values (1,000 replicates) are shown as percentages at the branch nodes. (B) The heatmap of ANI percentage identity for PeaFJ1 with other R. solanacearum strains. The number in the heatmap indicates different ANI percentage. [file Image_2.tif]

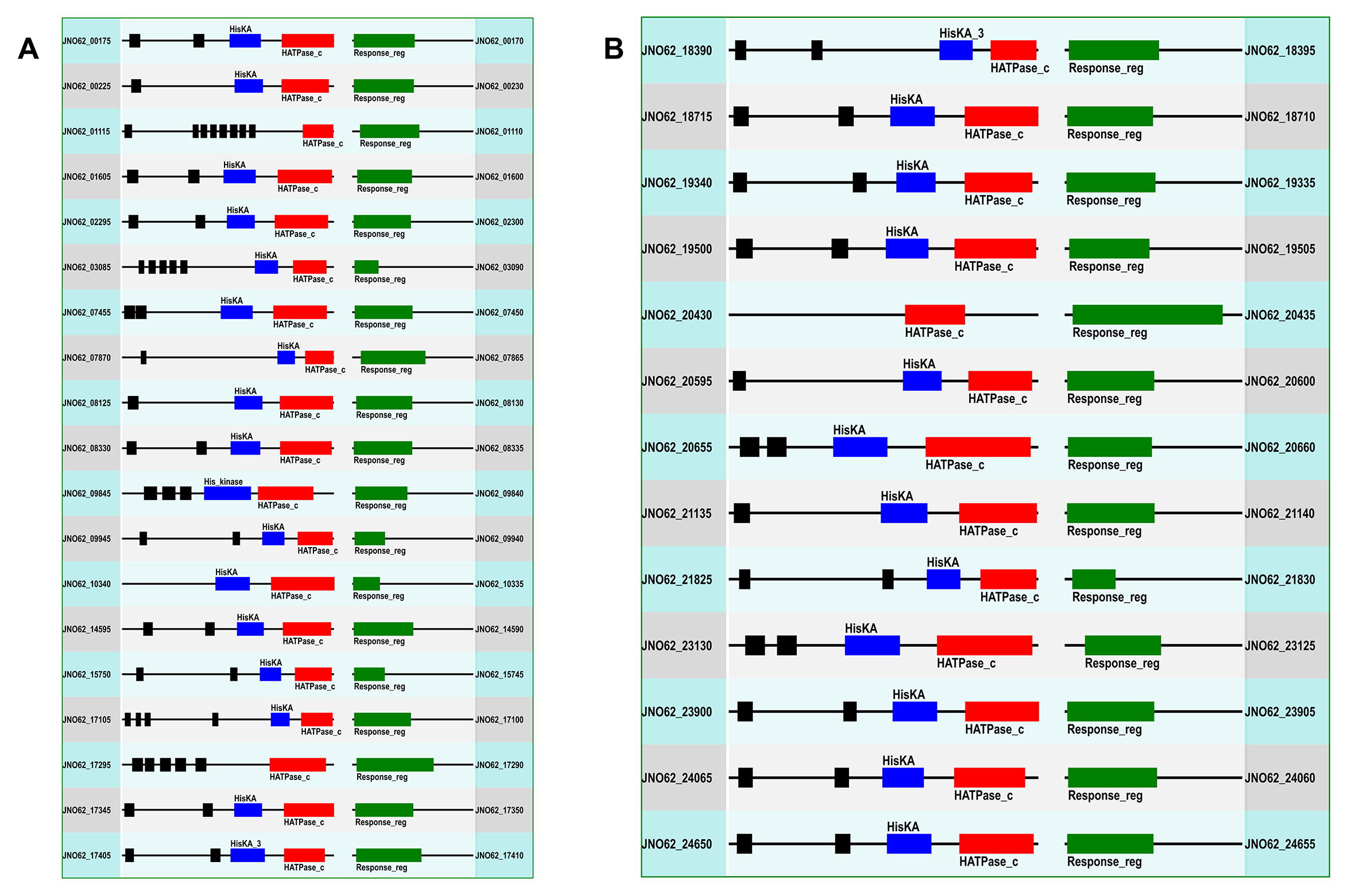

Supplement: Supplementary Figure 3 — Schematic diagram of two-component systems in R. solanacearum PeaFJ1. (A) The 19 TCSs in the chromosome. (B) The 13 TCSs in the megaplasmid. HisKA (blue): Phosphate receptor domain containing an H-box of conserved histidine sites capable of autophosphorylation. HATPase_c (red) is the intracellular catalytic domain of histidine kinase, responsible for the transfer of ATP phosphate groups to histidine. Response_reg (green): Response regulator (RR), response regulator protein. Black module: transmembrane helix. Numbers at both ends: gene ID. [file Image_3.tif]
